# Osteoporosis in brazilian patients awaiting knee arthroplasty

**DOI:** 10.1590/1413-785220172503167325

**Published:** 2017

**Authors:** David Sadigursky, Luiz Alberto Barretto, Diogo Maciel Vieira Lobão, Rogério Jamil Fernandes Carneiro, Paulo Oliveira Colavolpe

**Affiliations:** 1 Clínica Ortopédica Traumatológica (COT), Salvador, BA, Brazil; 2 Faculdade de Tecnologia e Ciências (FTC), Salvador, BA, Brazil

**Keywords:** Osteoporosis, Climacteric, Arthroplasty

## Abstract

**OBJECTIVE:**

The primary objective of this study was to determine the prevalence of osteoporosis and osteopenia prior to total knee arthroplasty (TKA) in female patients. As a secondary objective, we evaluated the incidence of hip fractures, types of drugs to treat osteoporosis and serum vitamin D levels.

**METHOD:**

This is a transversal, descriptive and observational study which evaluated 60 women above age 55 prior to total knee replacement.

**RESULTS:**

Mean patient age was 71.4 years. Osteoporosis was present in 16.7% of the sample and osteopenia in 15%. In the patients with osteoporosis, femur fracture (20%) was most frequent. Most of the group with osteopenia did not take any medication to treat this condition (55.6%), while most patients with osteoporosis took alendronate (30%) and 30% did not take any medication.

**CONCLUSION:**

The female population awaiting total knee replacement should be considered at risk for osteoporosis, confirming recent findings in the literature. ***Level of Evidence III, Control Case Study.***

## RESUMO

**OBJETIVO:**

O objetivo primário do estudo foi determinar a prevalência de osteoporose e osteopenia no pré-operatório de artroplastia total de joelho (ATJ) em pacientes do sexo feminino. Como objetivos secundários, avaliamos a incidência de fraturas de fêmur, o uso de medicações para o tratamento da osteoporose e os níveis da 25-OH vitamina D.

**MÉTODO:**

É um estudo transversal, descritivo e observacional. A amostra foi composta por 60 mulheres com idade acima de 55 anos, no climatério, em pré-operatório de ATJ.

**RESULTADOS:**

A média de idade foi de 71,4 anos. A osteoporose estava presente em 16,7% e a osteopenia em 15% da amostra estudada. Entre os pacientes com osteoporose, a fratura de fêmur foi a mais frequente (20%). A maioria do grupo com osteopenia não usava medicação para tratar essa afecção (55,6%), enquanto a maior parte dos pacientes com osteoporose usava alendronato (30%) e 30% não usavam nenhum medicamento.

**CONCLUSÃO:**

A população do sexo feminino aguardando artroplastia total de joelho deve ser considerada em risco de acometimento pela osteoporose, confirmando dados recentes da literatura. ***Nível de Evidência III, Estudo de Caso Controle.***

## INTRODUCTION

IBGE data from 1994 can be used to estimate that in Brazil there are 2.5 million osteoporotic individuals and 105,000 cases of hip fractures per year, resulting in a cost of approximately 630 million reais.^1^

In a population-based study, Pinheiro et al.^2^ demonstrated that smoking was the most important risk factor for osteoporosis in men. For women, the main factors are advanced age, early menopause, higher phosphorus intake, chronic use of benzodiazepines and family history of hip fracture after age 50 (first-degree relatives). Sedentary lifestyle, worsened quality of life and diabetes mellitus (DM) were considered common risk factors in both sexes. A prevalence study reported that higher body mass index (BMI) was associated with lower risk of disease involvement.^1^

Total knee arthroplasty (TKA) is a highly successful operation for treating degenerative changes of the knee such as osteoarthritis and demand for this procedure is growing worldwide due to aging populations and the need to preserve their quality of life.^3^ ^,^ ^4^ According to the National Institutes of Health Consensus, the most common long-term complications are aseptic loosening, pain and functional limitation, progressive bone loss, polyethylene wear and infection.^4^

Aseptic loosening leads to nearly half of indications for revision of primary arthroplasties.^5^ The cause of this complication is still the focus of much study and the current explanation is multifactorial, involving adaptive bone remodeling (stress shielding), micro-movement, high intra-articular pressure and individual susceptibility to microparticles.^6^ Furthermore, osteolysis is a factor that contributes to loosening of the implant and in some cases is related to osteoporosis.^7^

Some authors speculate that patients with osteoporosis have increased risk for periprosthetic fractures and consequently loosening of the implant due to the presence of micro-fractures and loss of contiguity in the bone tissue in this group.^4^ ^,^ ^8^

Domingues et al.^9^ found a 20.7% prevalence of osteoporosis and 37.9% for osteopenia in patients awaiting total hip arthroplasty. Additionally, the same study found a high prevalence of inadequate serum vitamin D levels, with only 16.6% of patients demonstrating levels considered normal (>30 ng/ml).

Based on the data above from the current literature, the present study aims to determine the prevalence of osteoporosis in patients undergoing TKA and also compares calcium and vitamin D levels, as well as preoperative densitometry findings. It is extremely important to understand the impact of this disease on women's health, since there are currently no studies in the literature correlating osteoporosis and TKA.

The objectives of the study were to determine the prevalence of osteoporosis prior to TKA in female patients and correlate levels of pain and functional capacity in patients with decreased bone mass in order to assess whether patients with osteoporosis and osteopenia demonstrate clinical profiles comparable to the population with normal bone density levels. The study also aimed to demonstrate if the involvement of osteoporosis had some direct or inverse correlation with the degree of functional limitation and pain prior to surgery.

## METHODS

This original study is cross-sectional, descriptive and observational. We assessed 60 patients over 55 years of age prior to TKA; only female patients were selected. Data were collected from july 2015 to february 2016.

This study was approved by the institutional review board under CAAE number 49258815.9.0000.5032. The subjects involved in this study signed a free and informed consent form.

Inclusion criteria were: diagnosis of osteoarthritis of the knee confirmed by radiography, scheduled TKA procedure and consent to participate in the study.

Exclusion criteria were: previous infection, congenital diseases, neoplasms, inflammatory arthritis, secondary osteoporosis, previous orthopedic surgery, hypothyroidism, decompensated DM and use of corticosteroids.

Bone mineral density (BMD) was measured by dual-energy x-ray absorptiometry (DEXA) in the femur and hip of all patients. Only the lower bone mass values were recorded in each component of the sample.

Osteoporosis was defined as a decrease in bone mass of at least 2.5 standard deviations from the mean young adult BMD (T-score < -2.5) and osteopenia was defined in patients with BMD values between -1 and -2.5 standard deviations from the mean peak value in young adults.^2^

A comparison of pain and functional impairment was established between patients with and without osteoporosis to establish correlations between the degree of osteoarthritis and decreased bone mass. Three questionnaires were used: the Western Ontario and McMaster Universities Osteoarthritis index (WOMAC),^10^ the Knee Society Score index (KSS),^11^ and the Visual Analog Pain Scale (VAS).^12^ The questionnaires were applied to all patients preoperatively. The Ahlback classification modified by Keyes et al. apud Galli et al.^13^ was used to establish the degree to which the knee joint is compromised in knee osteoarthritis. We also compared fracture involvement in the groups with osteoporosis, osteopenia and no changes in BMD in order to identify patterns that worsen quality of life in these patients.

The angular deviation of the lower limbs (LL) was assessed in all patients via physical examination and panoramic radiography. X-rays of all patients were taken from the front and profile views, as well as an axial view of the patella at 45° knee flexion, in addition to front and profile panoramic radiography; patients were classified as genu varum or genu valgum.

The variables analyzed were age, sex, degree of deformity, comorbidities, lifestyle habits (particularly smoking and alcoholism), serum level of 25-OH-D, ionic calcium, presence of osteoporosis and osteopenia according to BMD, use of medications to treat osteoporosis/osteopenia, hormone replacement therapy, vitamin D or calcium supplementation, BMI and previous fractures. 

The statistical analysis was conducted using Excel 2003 (Microsoft Corporation, Redmond, WA, USA) and SPSS 20.0 (IBM, New York, NY, USA) software. The tests were performed at a 5% significance level. The qualitative characteristics were described using absolute and relative frequencies and the quantitative measures were described in summary measures (mean, standard deviation, minimum and maximum). The quantitative characteristics were described according to the presence of osteoporosis and compared between patients with and without osteoporosis using Mann-Whitney tests.

## RESULTS

Table 1 describes the sample studied. The average age was 71.4 years with standard deviation (SD) of 6.9, minimum of 58 years and maximum of 85.

**Table 1:** Description of the sample.

Variable	N=60 (%)	Mean±SD (min-max)
Age (years)		71.4 ± 6.9 (58-85)
Ionic calcium		4.85 ± 0.31 (4.2-5.5)
Vitamin D (25-OH-cholecalciferol)		32 ± 7.12 (22-43)
Lifestyle factors		
Tobacco user	4 (6.7)	
Alcohol user	5 (8.3)	
Non-user of tobacco or alcohol	51 (85)	
Comorbidities		
High blood pressure	21 (35)	
Hypothyroidism	6 (10)	
Diabetes mellitus	11 (18.3)	
Dyslipidemia	6 (10)	
Heart failure	1 (1.7)	
History of breast cancer	1 (1.7)	
No comorbidities	26 (43.33)	

Mean ionic calcium was 4.85 with SD of 0.31; mean vitamin D (25-OH-cholecalciferol) was 32 with SD of 7.12. Four patients were smokers and five used alcohol. Table 1 shows the comorbidities found in each patient through the pre-procedure diagnostic questionnaire, medical records and physical examination. Hypertension was most frequent (35%), followed by DM (18.3%), hypothyroidism (10%) and dyslipidemia (10%). No other clinically relevant comorbidities were reported by the patients.

Table 2 below shows the presence of alterations in BMD. Osteoporosis was present in 10 of the individuals surveyed (16.7%) and osteopenia in 9 (15%).

**Table 2:** Changes in bone density in the sample.

Changes in bone density	N (%)
Osteoporosis	10 (16.7)
Osteopenia	9 (15)
Normal	41 (68.3)

Angular deviation in the lower limbs (genus valgum, genus varum) was compared in patients with osteopenia, osteoporosis, or without BMD changes. Genus valgum was detected in 70% of patients with osteoporosis and 55.6% of patients with osteopenia. Genus varum was the most common change (61%) in patients without changes in the BMD.

We also evaluated the BMI of patients in each group based on changes in BMD (osteoporosis, osteopenia and no bone changes). The group classified as having osteoporosis had a mean BMI of 27.3 (SD 2.9), the osteopenia group had mean BMI of 27.44 (SD 3.6) and the group with no changes in the densitometry had a mean BMI of 27.15 (SD 3.0).

Table 3 contrasts the bone density classification and fracture history of the patients. We can see that the group with osteoporosis had the most fractures, most frequently femur fracture (20%).

**Table 3:** History of fractures in each group.

Fracture	Group of patients according to changes in bone densitometry (mean±SD)			p* (95% CI)
	Osteoporosisn=10	Osteopenian=9	No bone changesn=41	
Spinal fractures (due to osteoporosis)	1 (10)	1 (11.1)	0	0.803 (0.796-0.811)
Fracture of proximal femur	2 (20)	0	1 (2.4)	
Fracture of distal radius	1 (10)	0	0	
No fractures	6 (60)	8 (88.9)	40 (97.6)	

*Pearson's chi-squared test with statistical significance if *p* is less than 0.05.

Table 4 shows the use of biphosphonates for prevention and/or treatment and also divides patients by osteoporosis, osteopenia and no bone alterations. Most of the osteopenia group did not use any anti-resorption medication (55.6%), while most of the patients with osteoporosis used alendronate or no medication (30%).

**Table 4:** Medications used in each group.

Variable	Group of patients according to changes in bone densitometry (%)		
	Osteoporosisn=10	Osteopenian=9	Normal BMDn = 41
Medication for osteoporosis			
None	3 (30)	5 (55.6)	41 (100)
Risedronate	2 (20)	2 (22.2)	0
Alendronate	3 (30)	1 (11.1)	0
Ibandronate	0	1 (11.1)	0
Zoledronic acid	2 (20)	0	0
Denosumab	0	0	0
Teriparatide	0	0	0
Hormone replacement	0	3 (33.3)	0
Vitamin D replacement	7 (70)	6 (66.7)	0

Table 5 describes pain levels according to VAS^12^ and functional capacity according to KSS^14^ and WOMAC^10^ in patients with osteoporosis, osteopenia and no bone changes. Note that there was no statistically significant difference for any scale in the preoperative period and that 100% of the evaluated patients with osteoporosis showed insufficient levels of 25-OH-cholecalciferol (reference value: <30 ng/mL).

**Table 5:** Comparative analysis of patients with osteoporosis, osteopenia and normal patients according to VAS, KSS and WOMAC scores*

	Group of patients according to changes in bone densitometry (mean±SD)			P* (95% CI)
	Osteoporosisn= 10	Osteopenian= 9	No bone changesn=41	
Visual Analog Scale				
Preoperative	8.2 ± 0.79	7.8 ± 0.9	7.8 ± 0.6	0.346 (0.337-0.355)
Knee society score				
Preoperative	44.1 ± 5.67	39.8 ± 5.3	42.6 ± 6.5	0.273 (0.265/0.282)
WOMAC score				
Preoperative	60.4 ± 7.15	59.7 ± 7.0	62.2 ± 7.7	0.356 (0.346/0.365)

*VAS = Visual Analog Pain Scale, Knee Society Score, Western Ontario and McMaster Universities osteoarthritis index. *Kruskal-Wallis test with statistical significance if *p* is less than 0.05.

## DISCUSSION

This is the first prevalence study that establishes a correlation between osteoporosis and TKA. Patients with osteoarthritis who need joint replacement are predominantly elderly women, a population with a high risk of developing osteoporosis,^15^ which justifies the selection of only women for this study. The selected age range coincides with menopause.

In this study we found that 31.7% of patients had decreases in bone mass and 16.7% had a confirmed diagnosis of osteoporosis from BMD and 15% had osteopenia. This value can be considered low in comparison with the study conducted by Pinheiro et al.^2^ on a sample of 4,332 patients in the metropolitan region of São Paulo, in which 33% of postmenopausal women showed osteoporosis of the lumbar spine or femur. Faisal-Cury and Zacchello^1^ found a 32.7% prevalence of osteoporosis in a group of 999 women above 49 years of age. We can therefore speculate that the difference between the results obtained may derive from differences in sample size.

Bisphosphonates are analogous to pyrophosphates and have an anti-resorptive action; they are among the most common therapeutic options for treating osteoporosis^16^ in order to prevent the occurrence of fractures. We found that 30% of patients with osteoporosis and 55.6% of patients with osteopenia were not taking bisphosphonate medication prior to the assessment. There was no statistically significant difference between the BMI of patients with osteoporosis, osteopenia and those with no changes in bone mass. The scores for the VAS,^12^ KSS,^11^ and WOMAC^10^ did not demonstrate a statistically significant difference between patients with and without osteoporosis.

Smoking has been established in the literature as a risk factor for osteoporosis. Only 6.7% of the population studied were smokers, but 80% of the patients who smoked had osteoporosis.^17^

Hormone replacement therapy is recommended for postmenopausal patients as the best way of preventing osteoporotic fractures, especially in the first years after last menstruation, although it should be used cautiously because of the increased risk of embolism and breast cancer.^15^ Based on this information, this study found that 33.3% of patients with osteopenia and no patients with unaltered bone mineral densitometry results used hormone replacement therapy. This finding may show insufficient monitoring for this disease in this group of women.

Calcitriol (1.25-dihidroxicolecalciferol) is the active form of vitamin D and plays a prominent role in bone metabolism, contributing to intestinal absorption of calcium and inhibiting resorption of the bone matrix.^18^ According to Glowacki et al.^19^ one of the most important factors contributing to its activation is exposure to sunlight. Serum values between 20-29 ng/mL indicate insufficient vitamin D, while levels below 20 ng/mL indicate an established deficiency of the hormone.^20^ A significant difference was seen in vitamin D levels among the groups with osteoporosis or osteopenia and no changes in bone mass. All patients diagnosed with osteoporosis showed insufficient levels, which indicates the significant influence this hormone deficiency plays in the presentation of the disease. 

One limitation of this study was the inclusion of the entire sample which fit the inclusion criteria, creating a convenience sample. A second limitation was the lack of a control group with patients without osteoarthritis matched for age and sex. The BMD only evaluated the hip and lumbar spine because these regions are most commonly included in assessment of the disease. Because this was a prevalence study, it was not possible to establish a causal relationship between exposure factors and outcomes.

The female population scheduled for TKA should receive special attention during the preoperative period since this group is notably susceptible to osteoporosis, in order to prevent complications such as osteoporotic and periprosthetic fractures and infection, which may lead to early loosening of the implant. We can infer that osteoarthritis does not appear to protect against osteoporosis, according to the same findings demonstrated by Domingues et al.^9^ if special attention is given to diagnosis in this group of patients and appropriate treatment is established.

## CONCLUSION

The prevalence of osteoporosis in the group studied was 16.7%, while the prevalence of osteopenia was 15%. The female population waiting for TKA should be considered at risk for involvement of osteoporosis in the studied population. The data we found resemble those found in the international literature.

No statistically significant difference was observed between pain levels and functional capacity among groups with osteoporosis, osteopenia, or with normal levels of bone mineral density.
